# Glycated Hemoglobin as a Predictor of Postoperative Delirium in Diabetic Patients Undergoing Noncardiac Surgery: A Retrospective Study

**DOI:** 10.3390/medicina61081474

**Published:** 2025-08-16

**Authors:** Mahir Bahceci, Ersel Gulec, Mediha Turktan, Zehra Hatipoglu, Dilek Ozcengiz

**Affiliations:** Department of Anesthesiology and Reanimation, Faculty of Medicine, Cukurova University, 01330 Adana, Turkey; mahir_bahceci@hotmail.com (M.B.); mediturktan@gmail.com (M.T.); hatipogluzehra@gmail.com (Z.H.); dilekozcengiz@gmail.com (D.O.)

**Keywords:** blood glucose, delirium, diabetes mellitus, general anesthesia, glycated hemoglobin, postoperative care

## Abstract

*Background and Objectives*: Diabetes is a known risk factor for postoperative delirium (POD); however, the relationship between the markers of glycemic control and the occurrence of POD in noncardiac surgery is not established. We initiated this pilot study to determine any possible associations between preoperative HbA1c levels and POD development; this will allow for larger, definitive studies to be designed and preliminary effect sizes to be established for future research. *Materials and Methods*: This retrospective pilot study included 78 patients with diabetes who underwent elective noncardiac surgery under general anesthesia between July 2020 and January 2021. We obtained the patients’ demographic data, medical history, surgical parameters, and preoperative HbA1c levels to determine the occurrence of POD (using CAM-ICU). Univariate and multivariate regression analyses were applied to check the leading associations for the development of POD. *Results*: POD was observed in seven patients (9.0%). The results of the preliminary multivariate analysis suggested that HbA1c may be associated with POD (OR, 2.96; 95% CI [1.34–6.52], *p* = 0.007); fasting blood glucose (OR, 1.04; 95% CI [1.01–1.07], *p* = 0.013); and duration of anesthesia (OR, 1.02; 95% CI [1.00–1.04], *p* = 0.019). The ROC analysis of HbA1c showed an optimal threshold of 7.4%, with a sensitivity of 91.5%, and a specificity of 85.7% in terms of predicting POD (AUC = 0.91, *p* < 0.001). *Conclusions*: Through this pilot study, we have provided evidence that leads to the assumption that preoperative HbA1c at, or above, 7.4% can result in an increased risk of delirium in diabetic patients who undergo noncardiac surgery. The findings of this study allow for the implementation of the proposed methodology and the collection of critical data necessary for the design of appropriately powered definitive trials.

## 1. Introduction

Postoperative delirium (POD) is one of the most important perioperative complications that influence patient outcomes and lengths of hospital stays, in addition to increasing healthcare costs. Postoperative delirium is a clinical condition characterized by acutely altered degrees of consciousness and cognitive dysfunction, with an attendant deficiency in attention [1,2]. Its occurrence in surgical patients ranges between 5 and 87% and higher in patients with diabetes mellitus (DM) [1,3,4,5,6,7,8]. As the prevalence of diabetes in surgical populations increases, the specific risk factors for targeted prevention need to be better understood.

Glycated hemoglobin (HbA1c) is the standard long-term marker of glycemic control in diabetic patients and correlates with average glucose concentrations over the preceding 2–3 months [9]. More recent evidence seems to indicate that HbA1c can predict POD independently from the diagnosis of DM alone [10,11]. However, studies on the predictive value of HbA1c for POD in patients with diabetes undergoing noncardiac surgery are scarce; existing studies have not determined the specific thresholds that are easily applicable in clinical settings.

Most current studies in the literature focus on the cardiac surgery population or do not separate or differentiate the specific influence of HbA1c levels on POD outcomes from the more general aspects of diabetes management. This research gap constitutes a major limitation because the noncardiac surgical population could have different risk profiles and outcomes compared to cardiac surgery patients. Furthermore, the absence of recommended thresholds for HbA1c in relation to POD risk stratification affects the applicability of the available research in practice.

Due to limited research that specifically examined HbA1c thresholds for the risk of POD in noncardiac surgery, this retrospective pilot study was designed with the following objectives: we aimed to determine preliminary effect sizes to guide power calculations for future definitive studies; assess whether the proposed methodology and data collection procedures were feasible; explore possible confounding variables and other considerations that could affect future research; provide preliminary evidence useful for funding applications for larger trials; and develop a clinical threshold for HbA1c that can be further tested in prospective studies, an essential objective of this study. Therefore, this study was designed to explore the association between preoperative HbA1c levels and the incidence of POD in diabetic patients undergoing elective noncardiac surgery.

## 2. Materials and Methods

### 2.1. Study Design and Ethics

This retrospective pilot study involved 78 diabetic patients who underwent elective noncardiac surgery with general anesthesia from July 2020 to January 2021. This study received approval from the local institutional Clinical Research Ethics Committee (protocol code: 41/107, January 2021) and complied with the Good Clinical Practice guidelines and the Declaration of Helsinki [12]. Since this pilot study was retrospective in nature, the requirement for IRB consent was waived.

### 2.2. Study Population and Sample Size

**Figure 1:**
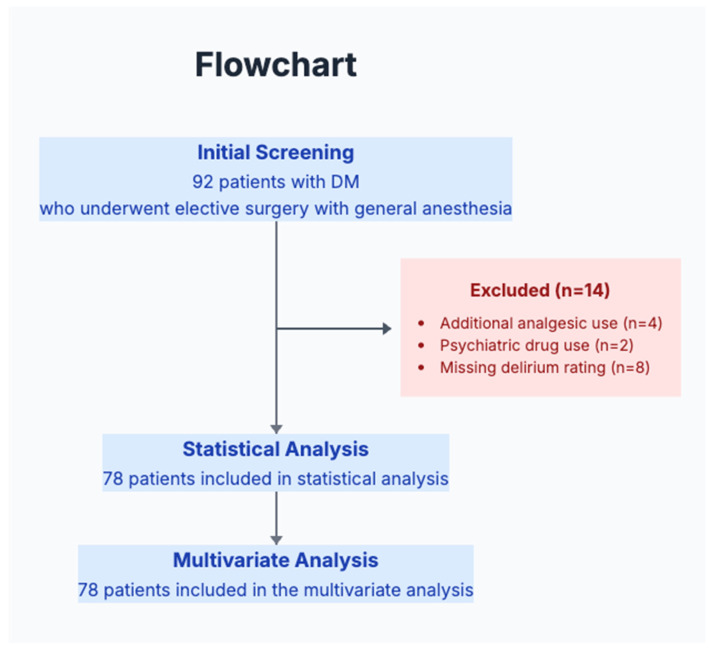
Study flowchart.

As a pilot study, our goal was to recruit a convenient sample to determine preliminary effect sizes rather than to test definitive hypotheses. Retrospective data from diabetic patients who underwent elective surgery with general anesthesia between July 2020 and January 2021 were analyzed. The literature on this topic and clinical experience led us to expect that the incidence of POD in diabetic patients is approximately 8–15%. A final sample size of 78 patients was determined by the study period and available resources. A total of 78 out of 92 possible patients met the eligibility criteria, representing an inclusion rate of 85%, which demonstrates good feasibility for future studies (Figure 1).

### 2.3. Inclusion and Exclusion Criteria

Patients were included in the study if they met the following criteria: age 18 years or older, a diagnosis of diabetes mellitus, underwent elective noncardiac and nonintracranial surgery, received general anesthesia, and had their preoperative HbA1c levels available within 3 months of surgery. Patients were excluded if they had cognitive impairment or a history of delirium, psychiatric disorders or substance abuse, emergency surgery, or cardiac or intracranial procedures. Patients with incomplete medical records and insufficient data for assessing delirium were also excluded.

### 2.4. Gathering Information

The data collection process focused on variables previously identified in the literature that contribute to the risk of POD. These included demographic information, age, gender, and body mass index. Medical history included conditions such as hypertension, stroke, coronary heart disease, chronic lung disease, renal dysfunction, and malignancy. More information on diabetes management was documented in terms of the type of antidiabetic medication (insulin, oral medications, or combination therapy). The surgical parameters were the type and duration of the surgery, as well as the duration of the anesthesia. The laboratory values of HbA1c, fasting blood glucose, hemoglobin, and hematocrit were also collected.

### 2.5. Assessment of the Outcomes

Primary Outcome: Assessing the incidence of POD using the Confusion Assessment Method for the Intensive Care Unit (CAM-ICU) performed at discharge from the postoperative care unit [13].

Secondary Outcomes: The identification of an HbA1c threshold for the prediction of POD, the evaluation of other potential risk factors, and the evaluation of feasibility metrics to inform future study designs.

### 2.6. Pilot-Study-Specific Assessments

In addition to the clinical outcomes, several feasibility parameters were evaluated. These included recruitment feasibility, which measured the proportion of eligible patients identified during the initial screening, and data completeness, which refers to the availability of all the required variables. We recorded the time spent with each patient. Finally, the assessment barriers, specifically, the challenges encountered in implementing CAM-ICU evaluations, were noted.

### 2.7. Comorbidity Assessment

The Charlson Comorbidity Index (CCI) was used to quantify the overall comorbidity burden [14]. Surgical severity was classified according to the established operative severity definitions [15].

### 2.8. Statistical Analysis

Statistical analyses were performed using IBM SPSS 26. Descriptive statistics included frequencies and percentages of categorical variables and means (SD) or medians (IQR) for continuous variables. Normality was assessed using the Shapiro–Wilk test.

Patients were grouped based on whether they developed POD. The Mann–Whitney U-test was performed to compare non-normally distributed continuous variables; the independent t-test was used to compare normally distributed variables. Categorical data were analyzed using the chi-squared test or Fisher’s exact test, as appropriate. Univariate and multivariate logistic regression analyses were performed to establish preliminary odds ratios and confidence intervals. A receiver operating characteristic (ROC) curve analysis was performed to detect the optimal cutoff point of HbA1c using the Youden index. The model’s fit was evaluated using the Hosmer–Lemeshow test. Statistical significance was set at *p* < 0.05.

## 3. Results

### 3.1. Feasibility Outcomes

Of the 92 potentially eligible patients identified during the study period, the data of 78 (85%) patients were complete, as required for the analysis, demonstrating good feasibility for future prospective studies. The review of the chart required approximately 15 min per patient. HbA1c values were available for all patients within 3 months of surgery. The main reason for patient exclusion was missing delirium assessment notes (*n* = 8, 57% of exclusions), indicating the need for a standardized assessment protocol in future studies.

### 3.2. Patient Characteristics and Incidence of POD

Seven patients (9.0%) developed POD, consistent with the rates reported in diabetic surgical populations. The patient demographics and clinical characteristics are presented in Table 1. The mean age of the patients was 57.9 ± 9.9 years and 46% of the patients were female.

### 3.3. Preliminary Clinical Associations

Patients who developed POD had significantly higher mean HbA1c levels than those without POD (9.0 ± 2.2% vs. 6.6 ± 1.1%, *p* = 0.027). When stratified by glycemic control (HbA1c < 7% vs. ≥7%), patients with poorly controlled diabetes showed a significantly higher risk of POD (*p* = 0.003). Patients with POD underwent longer surgical procedures (187.1 ± 71.6 vs. 119.3 ± 78.5 min) and a longer duration of anesthesia (200.4 ± 71.7 vs. 129.9 ± 80.0 min). The variables associated with POD in the preliminary univariate analysis included the duration of anesthesia, duration of surgery, HbA1c, fasting blood glucose, and the use of oral antidiabetic drugs (see Table 2).

We recognized that there was a limited number of events (*n* = 7) relative to the number of variables; therefore, we performed an exploratory multivariate analysis that included the strongest predictors. The variables associated with POD included HbA1c, fasting blood glucose, and anesthesia duration (see Table 2).

**Figure 2:**
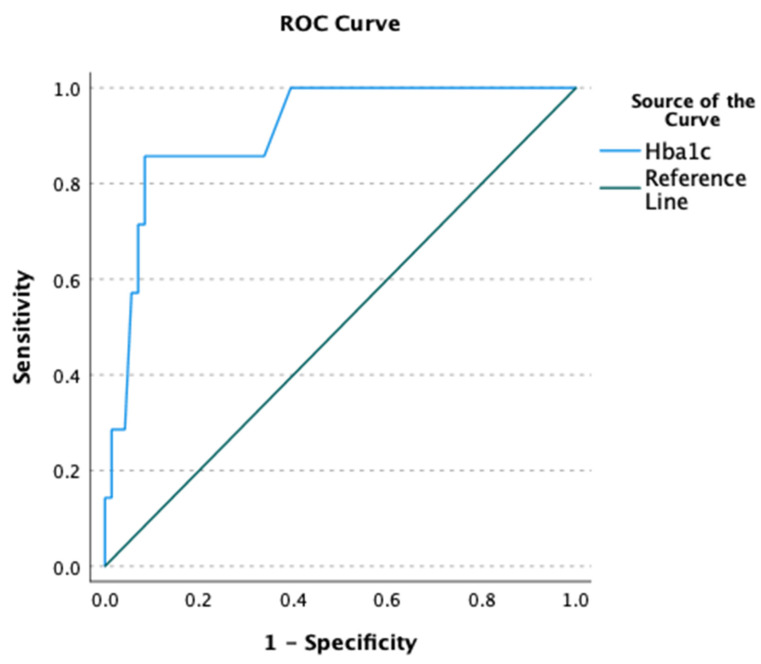
ROC curve for HbA1c and postoperative delirium. AUC = 0.91 (95% CI: 0.81–1.00), *p* < 0.001. Optimal cutoff: 7.4% (sensitivity 91.5%; specificity 85.7%).

Via the ROC curve analysis, we identified an HbA1c cutoff of 7.4% with optimal performance characteristics, demonstrating a sensitivity of 91.5%, specificity of 85.7%, and area under the curve of 0.91 (95% CI [0.81–1.00], *p* < 0.001) (Figure 2).

### 3.4. Post Hoc Power Analysis and Sample Size Calculations for Future Studies

Because there were only seven cases of delirium against three predictors in the multivariate logistic regression, we performed a post hoc analysis to estimate the potential risk of overfitting. The events-per-variable (EPV) ratio was 2.3 (seven events ÷ three predictors), significantly below the recommended minimum of 10 EPV for stable logistic regression estimates. A low EPV ratio is an indicator of an increased risk toward overfitting the model, thereby causing unstable effect estimates and for the performance metrics to be overly optimistic.

Via the post hoc power analysis, we showed that this study has a statistical power of 78.2% with an 0.05 alpha level for the main analysis using HbA1c (odds ratio = 2.96; Cohen’s d = 0.598), which is very close to the usual standard of 80%. However, the secondary predictors, fasting blood glucose and duration of anesthesia, were notably underpowered at 5.1% and 5.0%, respectively.

For a multivariate logistic regression model with three predictors to satisfy the recommended minimum of 10 events per variable (that is, approximately 30 total events), and considering a POD incidence rate of 9.0%, about 334 patients would be required. Using this sample size, there is an 80% probability of detecting an odds ratio of 2.0 or more for HbA1c, with a significance level of α = 0.05, indicating clinical relevance.

## 4. Discussion

Based on the findings of this pilot study, we suggest that a preoperative HbA1c level of 7.4% or higher may increase the risk of POD in diabetic patients undergoing noncardiac surgery. Through our exploratory analysis of this study, we indicated a significant odds ratio of 2.96, highlighting the need for more in-depth research. The proposed HbA1c threshold exhibited an AUC of 0.91, indicating a strong predictive capability. However, exercising caution regarding these results is necessary because of the small sample size of this pilot study and the very few outcome events, suggesting potential overfitting. The required guideline of 10 events per model variable was not met, which increases this risk. Therefore, the 7.4% threshold is not ready for clinical use; however, it does offer a testable hypothesis for further studies. Unlike larger studies, where high AUCs are significant, even large-scale cardiac surgery models show AUCs from fair to good (0.61–0.93), with high values generally decreasing after external validation [16].

In this study, we focused on HbA1c, an indicator of long-term blood sugar control, as elevated levels are associated with various postoperative complications [17]. However, the literature suggests that the timing of hyperglycemia in noncardiac surgeries is especially important. In a recent study, distinguishment between the chronic and acute glycemic effects in more than 23,000 patients with diabetes who underwent noncardiac surgery was possible. The investigators found that acute preoperative hyperglycemia (fasting glucose > 140 mg/dL or random glucose > 180 mg/dL within 24 h after surgery) was significantly associated with an increased risk of POD (hazard ratio 1.33). In contrast, chronic hyperglycemia, defined as HbA1c >6.5%, was not independently associated with delirium in their adjusted model [18]. This finding challenges the notion of HbA1c being the primary risk factor for POD, suggesting that acute perioperative glycemic surges may be more influential. Various studies indicate that intraoperative hyperglycemia (≥150 mg/dL) is strongly associated with POD, with an odds ratio of 3.86 [19]; higher random blood glucose levels in patients in the PACU are significantly associated with delirium [20].

The difference between our pilot findings on HbA1c and those of larger studies on acute hyperglycemia may highlight a two-part delirium mechanism. Chronic hyperglycemia, indicated by elevated HbA1c levels, predisposes patients to increased cerebral vulnerability through neuroinflammation and endothelial dysfunction. The acute hyperglycemic surge in surgical stress then triggers the vulnerable brain to develop delirium [21]. Our pilot study focused on diabetic patients with elevated HbA1c levels, possibly creating a group already at risk. For these individuals, HbA1c alone may indicate an overall risk, whereas, in a diverse population, acute hyperglycemia could be a stronger predictor.

The key limitation of this pilot study was the need to rely on a single preoperative biomarker. Effective perioperative glucose control, including the management of glycemic variability and hypoglycemia, is vital for patient outcomes. Evidence indicates that fluctuations in blood glucose levels can be as harmful as consistently high levels. A 2021 study of 705 patients who underwent cardiac surgery found that intraoperative glycemic variability (GV), measured by the coefficient of variation, was a strong predictor of POD. Patients in the top GV quartile had a 3.65 odds ratio for developing delirium compared to those in the lowest quartile [22]. Another study found that, in patients with DM who underwent cardiac surgery, higher levels of GV metrics, such as the mean amplitude of glycemic excursions and glycemic lability index, were associated with an increased chance of developing POD [21].

This risk involves high and low glucose levels, forming a U-shaped relationship [23]. One study linked hypoglycemia with a 2.78 odds ratio for delirium in patients with DM. Tight glucose control, which increases the risk of hypoglycemia, is associated with higher rates of delirium [22]. Glucose management should focus on stability rather than simply lowering blood sugar levels. Avoiding hyperglycemia and strategies that cause wide glucose fluctuations are crucial for brain health. Our pilot study could not capture these perioperative glycemic dynamics.

Elevated preoperative HbA1c is a biomarker, but not a direct cause, of a brain that is vulnerable to surgery-induced delirium. This supports the “two-hit” model of delirium, in which a predisposing factor decreases brain resilience, leading to susceptibility to subsequent insults [19]. Chronic hyperglycemia, indicated by HbA1c levels, damages the brain microenvironment over time by forming advanced glycation end-products, leading to systemic inflammation [23]. This primes brain immune cells such as microglia. Surgical trauma, as a “second hit”, releases proinflammatory cytokines, such as TNF-α, IL-1β, and IL-6, triggering severe neuroinflammation. This response causes delirium, synaptic dysfunction, neuronal apoptosis, and altered neurogenesis [24]. Diabetes is primarily a vascular disease characterized by chronic high blood sugar levels that cause endothelial dysfunction and blood–brain barrier (BBB) damage [23]. This increases BBB permeability and weakens brain defenses. A compromised BBB allows for inflammatory mediators, neurotoxic substances, and immune cells to enter the brain, worsening inflammation and disrupting neuronal function [21]. This inflammation can disrupt neurotransmitter systems, particularly the cholinergic system, which is crucial for attention and cognition. Systemic inflammation is linked to reduced acetylcholine levels in the brain; butyrylcholinesterase, which regulates acetylcholine levels, may contribute to POD development [25]. Therefore, the finding that HbA1c predicts POD in a diabetic population serves as a direct clinical validation of the two-hit model. This demonstrates that the severity of the chronic “first hit” (chronic dysglycemia) is a key determinant of the brain’s ability to withstand the acute “second hit” of surgery. This elevates the discussion from a simple risk factor analysis to a clinical confirmation of the core pathophysiological theory of delirium.

Our analysis identified secondary associations that are worthy of discussion. A longer anesthesia duration is linked to POD, which is consistent with extensive evidence. This complex, robust risk factor is documented in numerous studies and meta-analyses [26,27,28,29]. Anesthesia duration likely indicates multiple underlying issues, such as surgical complexity, tissue trauma, inflammation, and extended anesthetic exposure, all of which increase the amount of stress that the central nervous system is under [26,30,31,32].

Our pilot study did not find a significant link between advanced age and POD, deviating from most of the delirium literature, where age is a key non-modifiable risk factor [33,34]. This is likely due to the limited sample size and not having any evidence against the importance of age as a risk factor.

The univariable analysis showed that oral antidiabetic medications had a protective effect, which contradicts previous research associating them with a higher risk of POD in cardiac surgery [10]. This difference may be due to confounding by indication and the effects of drugs. Insulin users generally have more severe diabetes, which poses a higher risk. Metformin, a common oral antidiabetic, may offer neuroprotection, reducing the risk of delirium (pooled relative risk: 0.71) [35]. Our cohort likely included many metformin users, which may have skewed the results. This finding suggests that metformin has neuroprotective potential in perioperative care, which warrants further research.

### 4.1. Limitations

Since this study was conducted at a single tertiary referral center, the results may not be directly applicable to other populations or institutions. Variations from studies in the literature may arise in terms of our unique perioperative and social approach, and the impact of the strain of the COVID-19 pandemic on local healthcare during the study period, in addition to the focus on diabetic patients. This pilot study’s findings are limited by its retrospective design, which risks selection bias and depends on the quality of the clinical documentation. During data collection, the main reason for patient exclusion was the incomplete documentation of delirium assessment, highlighting the challenges of retrospective delirium research. The limited number of events weakens the statistical power of the analyses and raises the possibility of overfitting in the regression model. Therefore, the effect estimates from this analysis are preliminary and require validation in larger studies. A single POD assessment at PACU discharge may underestimate the outcomes because delirium is an acute syndrome with fluctuating symptoms. This single assessment often misses delayed-onset delirium, possibly explaining the lower observed incidence (9.0%). The ESAIC guidelines recommend using a validated screening tool daily for at least three postoperative days to accurately determine the incidence of POD [36]. We could not fully account for all the potential confounding variables that could affect the risk of POD. The unmeasured factors in this study included anesthetic agents; anesthesia depth; intraoperative hemodynamic stability; hypotension; postoperative pain management; total opioid dosage; and glycemic variability measures. These factors may influence the observed associations.

### 4.2. Future Research

Notwithstanding this pilot study’s limitations, we provide valuable clinical insights that will be useful for future studies. The 7.4% HbA1c threshold has not yet been clinically validated; however, it highlights chronically high HbA1c levels as a key indicator of POD risk. This aligns with the recommendations of professional societies, such as the STS and JBDS, to include preoperative HbA1c screening in surgical risk assessments [37]. However, the field currently lacks consensus on a universal HbA1c threshold for postponing elective surgery, with recommendations varying widely from >7.0% to >8.5% [38]. Our study suggests that this risk may increase slightly above the standard diabetic target of 7.0%. The key clinical takeaway is to implement a risk-stratified care pathway rather than a strict HbA1c cutoff for surgery in patients with DM. An elevated preoperative HbA1c level signals that the patient is at increased risk, prompting proactive interventions. From this perspective, HbA1c can be effectively utilized within prehabilitation programs to optimize a patient’s condition for surgery. This shifts the focus from a simple “go/no-go” surgery decision to “How can we best mitigate this risk?”. This pathway involves increased perioperative vigilance, intensive glucose monitoring, and the use of evidence-based non-pharmacological delirium prevention methods such as optimizing mobility, sleep, hydration, and sensory input.

This preliminary study provides important information for designing a larger trial to confirm these findings. This highlights the need for a prospective multicenter study to ensure sufficient statistical power, minimize bias, and enhance generalizability. Data collection should focus on comprehensive glycemic profiling, which includes measuring preoperative HbA1c levels and admission blood glucose, and continuously monitoring glucose both during and after surgery. This approach aims to analyze chronic and acute hyperglycemia and glycemic fluctuations. Additionally, a standardized tool should be employed to assess the incidence and various types of POD at least once daily for three–five days following surgery. It is also crucial to control for potential confounding factors by collecting data on patient demographics; comorbidities; cognitive and functional status; surgical parameters; hemodynamic status; and postoperative pain management. This future study should incorporate mechanistic sub-studies that collect biological samples to evaluate inflammatory biomarkers and endothelial dysfunction, thereby investigating the relationship between dysglycemia, neuroinflammation, and delirium. Finally, future trials should explore whether interventions, such as metformin or intranasal insulin, can effectively reduce the risk of POD in patients with DM.

## 5. Conclusions

In conclusion, through this pilot study, we offer vital insights into the relationship between preoperative HbA1c levels and the risk of POD in patients with DM undergoing noncardiac surgery. The findings suggest that an HbA1c threshold of 7.4% may indicate a threefold increase in the odds of developing POD, emphasizing the need for glycemic control in this vulnerable population. However, the limited sample size of this study and the small number of observed outcomes mean that caution should be taken when generalizing these results. Despite this study’s limitations, the strong predictive capability of HbA1c highlights its potential as a biomarker for identifying at-risk patients. Future research is imperative to validate these preliminary findings in larger multicenter trials, which should include comprehensive glycemic profiles and standardized assessment protocols to improve our understanding of the mechanisms underlying POD. Incorporating HbA1c screening into preoperative evaluations could enhance patient-specific risk stratification and inform targeted interventions aimed at reducing the incidence of POD, thereby improving overall surgical outcomes.

## Figures and Tables

**Table 1:** Univariable analysis and characteristics of the study population.

	POD (−) (*n* = 71)	POD (+) (*n* = 7)	Total	*p*-Value	*p*-Value ^§^
Anesthesia duration (min)	110 (70–150)	225 (162–255)	135.5 ± 82.2	**0.024**	**0.040**
Surgery duration (min)	100 (65–140)	210 (150–240)	124.7 ± 80.7	**0.026**	**0.044**
HbA1c (%)	6.6 ± 1.1	9.0 ± 2.2	6.8 ± 1.4	**0.027**	**0.005**
<7%	53 (74.6)	1 (14.3)	54 (69.2)		
≥7%	18 (25.4)	6 (85.7)	24 (30.8)	**0.003**	**0.003**
Fasting blood glucose (mg/dL)	128 (111–158)	194 (182–251)	142.8 ± 45.9	**0.008**	**0.005**
Number of blood transfusions	0 (0–0)	0 (0–1)	1.9 ± 0.7	0.069	0.228
Hb (g/dL)	12.6 ± 2.0	11.5 ± 1.5	12.5 ± 2.0	0.170	0.157
Hematocrit (%)	37.4 ± 5.7	33.5 ± 4.1	37.0 ± 5.7	0.087	0.095
BUN (mg/dL)	14.9 (12.6–19.0)	19.0 (12.5–27.7)	17.8 ± 12.5	0.441	0.243
Creatine (mg/dL)	0.8 (0.7–1.1)	1.2 (0.6–1.3)	1.1 ± 1.4	0.358	0.657
AST (U/L)	19.9 ± 5.4	19.0 ± 6.0	19.8 ± 5.5	0.689	0.684
ALT (U/L)	20.1 ± 7.9	19.0 (15.0–32.0)	20.8 ± 7.9	0.649	0.625
Sodium (mmol/L)	138.9 ± 2.8	140 ± 2.9	139.0 ± 2.8	0.555	0.314
Potassium (mmol/L)	4.4 (4.2–4.7)	4.2 (4.1–4.9)	4.4 ± 0.5	0.827	0.970
Age (yr)	57.8 ± 10.0	59.4 ± 9.9	57.9 ± 9.9	0.677	0.673
CCI	3 (2–4)	4 (2–4)	3 (2–4)	0.247	0.213
BMI (kg/m^2^)			29.3 ± 5.0		
Normal	11 (15.5)	4 (57.1)	15 (19.2)		0.058
Overweight	28 (39.4)	2 (28.6)	30 (38.5)		0.082
Obese	32 (45.1)	1 (14.3)	33 (42.3)	0.052	**0.036**
Gender					
Female	44 (62.0)	2 (28.6)	46 (59.0)	0.116	
Male	27 (38.0)	5 (71.4)	32 (41.0)		0.107
Alcohol use					
Yes	2 (2.8)	1 (14.3)	3 (3.8)	0.249	0.177
No	69 (97.2)	6 (85.7)	75 (96.2)		
Smoking					
Yes	22 (31.0)	1 (14.3)	23 (29.5)	0.667	0.372
No	49 (69.0)	6 (85.7)	55 (70.5)		
Antidiabetic treatment					
Insulin	13 (18.3)	3 (42.9)	16 (20.5)		0.345
Oral antidiabetic	53 (74.6)	1 (14.3)	54 (69.2)	**0.002**	**0.014**
Both	4 (5.6)	2 (28.6)	6 (7.7)		0.676
None	1 (1.4)	1 (14.3)	2 (2.6)		0.047
Antidiabetic medication use					
Yes	41 (57.7)	5 (71.4)	46 (59.0)	0.694	0.488
No	30 (42.3)	2 (28.6)	32 (41.0)		
Additional comorbidity					
None	25 (35.2)	2 (28.6)	27 (34.6)		0.561
Comorbidity	29 (40.8)	2 (28.6)	31 (39.7)		0.886
Multiple comorbidities	17 (23.9)	3 (42.9)	20 (25.6)	0.606	0.413
ASA					
Score 2	65 (91.5)	5 (71.4)	70 (89.7)		
Score 3	6 (8.5)	2 (28.6)	8 (10.3)	0.149	0.118
Surgical severity					
Minor	26 (36.6)	1 (14.3)	27 (34.6)		0.243
Moderate	33 (46.5)	3 (42.9)	36 (46.2)		0.468
Major	8 (11.3)	1 (14.3)	9 (11.5)		0.423
Major+	4 (5.6)	2 (28.6)	6 (7.7)	0.147	0.055
Postoperative analgesics					
Tramadol	32 (45.1)	1 (14.3)	33 (42.3)		0.127
Tramadol + paracetamol	18 (25.4)	1 (14.3)	19 (24.4)		0.690
Morphine	21 (29.6)	5 (71.4)	26 (33.3)	0.114	0.073

^§^ Univariable analysis was used. *p*-values are in bold if <0.05. ASA, American Society of Anesthesiologists score; AST, aspartate aminotransferase; ALT, alanine aminotransferase; BUN, blood urea nitrogen; CCI, Charlson Comorbidity Index score; Hb, hemoglobin.

**Table 2:** Logistic regression analysis of independent risk factors related to POD.

	Univariate Analysis			Multivariate Analysis		
Variable	OR	CI %95	*p*-Value	Adjusted OR	CI %95	*p*-Value
HbA1c (%)	2.19	1.27–3.78	0.005	2.96	1.34–6.52	0.007
Blood glucose (g/dL)	1.02	1.01–1.04	0.005	1.04	1.01–1.07	0.013
Anesthesia duration (min)	1.01	1.00–1.02	0.039	1.02	1.00–1.04	0.019
Surgery duration (min)	1.01	1.00–1.02	0.043	-	-	-
Oral antidiabetic drugs	0.038	0.003–0.511	0.014	-	-	-

OR: odds ratio; CI 95%: 95% confidence interval.

## Data Availability

Owing to ethical constraints, the corresponding author can provide the study data upon request.

## Abbreviations

The following abbreviations are used in this manuscript:

AUC	Area Under the Curve
BBB	Blood–Brain Barrier
CAM-ICU	Confusion Assessment Method for the Intensive Care Unit
CCI	Charlson Comorbidity Index
DM	Diabetes Mellitus
ESAIC	European Society of Anaesthesiology and Intensive Care
GV	Glycemic Variability
HbA1c	Glycated Hemoglobin
IQR	Interquartile Range
JBDS	Joint British Diabetes Societies
OR	Odds Ratio
POD	Postoperative Delirium
ROC	Receiver Operating Characteristic
SD	Standard Deviation
SPSS	Statistical Package for the Social Sciences
STS	Society of Thoracic Surgeons
